# Augmented Reality Meets Artificial Intelligence in Robotics: A Systematic Review

**DOI:** 10.3389/frobt.2021.724798

**Published:** 2021-09-22

**Authors:** Zahraa Bassyouni, Imad H. Elhajj

**Affiliations:** Vision and Robotics Lab, Department of Electrical and Computer Engineering, American University of Beirut, Beirut, Lebanon

**Keywords:** robotics, augmented realitiy, artificial intelligence, planning, learning, perception

## Abstract

Recently, advancements in computational machinery have facilitated the integration of artificial intelligence (AI) to almost every field and industry. This fast-paced development in AI and sensing technologies have stirred an evolution in the realm of robotics. Concurrently, augmented reality (AR) applications are providing solutions to a myriad of robotics applications, such as demystifying robot motion intent and supporting intuitive control and feedback. In this paper, research papers combining the potentials of AI and AR in robotics over the last decade are presented and systematically reviewed. Four sources for data collection were utilized: Google Scholar, Scopus database, the International Conference on Robotics and Automation 2020 proceedings, and the references and citations of all identified papers. A total of 29 papers were analyzed from two perspectives: a theme-based perspective showcasing the relation between AR and AI, and an application-based analysis highlighting how the robotics application was affected. These two sections are further categorized based on the type of robotics platform and the type of robotics application, respectively. We analyze the work done and highlight some of the prevailing limitations hindering the field. Results also explain how AR and AI can be combined to solve the model-mismatch paradigm by creating a closed feedback loop between the user and the robot. This forms a solid base for increasing the efficiency of the robotic application and enhancing the user’s situational awareness, safety, and acceptance of AI robots. Our findings affirm the promising future for robust integration of AR and AI in numerous robotic applications.

## Introduction

Artificial intelligence (AI) is the science of empowering machines with human-like intelligence (Nilsson, 2009). It is a broad branch of computer science that mimics human capabilities of functioning independently and intelligently (Nilsson, 1998). Although AI concepts date back to the 1950s when Alan Turing proposed his famous Turing test (Turing, 1950), its techniques and algorithms were abandoned for a while as the computational power needed was still insufficient. Recently, the advent of big data and the Internet of Things (IoT), supercomputers, and cheap accessible storage have paved the way for a long-awaited renaissance in artificial intelligence. Currently, research in AI is involved in many domains including robotics (Le et al., 2018; Gonzalez-Billandon et al., 2019), natural language processing (NLP) (Bouaziz et al., 2018; Mathews, 2019), and expert systems (Livio and Hodhod, 2018; Nicolotti et al., 2019). It is becoming ubiquitous in almost every field that requires humans to perform intelligent tasks like detecting fraudulent transactions, diagnosing diseases, and driving cars on crowded streets.

Specifically, in the field of robotics, AI is optimizing a robot’s autonomy in planning tasks and interacting with the world. The AI robot offers a greater advantage over the conventional robot that can only apply pre-defined reflex actions (Govers, 2018). AI robots can learn from experience, adapt to an environment, and make reasonable decisions based on their sensing capabilities. For example, research is now leveraging AI’s learning algorithms to make robots learn the best path to take for different cases (Kim and Pineau, 2016; Singh and Thongam, 2019), NLP for an intuitive human-robot interaction (Kahuttanaseth et al., 2018), and deep neural networks to develop an understanding of emotional intents in human-robot interactions (HRI) (Chen et al., 2020a; Chen et al., 2020b). Computer vision is also another field of AI that has enhanced the perception and awareness of robots. It combines machine learning with image capture and analysis to support robot navigation and automatic inspection. This ability of a robot to possess self-awareness is facilitating the field of HRI (Busch et al., 2017).

The field of robotics has also benefited from the rising technology of augmented reality (AR). AR expands a user’s physical world by augmenting his/her view with digital information (Van Krevelen and Poelman, 2010). AR devices are used to support the augmented interface and are classified into eye-wear devices like head-mounted displays (HMD) and glasses, handheld devices like tablets and mobile phones, and spatial projectors. Two other extended reality (XR) technologies exist that we need to distinguish from AR, and they are virtual reality (VR) and mixed reality (MR). VR is a system that, compared to AR which augments information on a live view of the real world, simulates a 3D graphical environment totally different from the physical world, and enables a human to naturally and intuitively interact with it (Tzafestas, 2006). MR combines AR and VR, meaning that it merges physical and virtual environments (Milgram and Kishino, 1994). Recently, the research sector witnessed a booming activity of integrating augmented reality in supporting robotics applications (Makhataeva and Varol, 2020). These applications include robot-assisted surgery (RAS) (Pessaux et al., 2015; Dickey et al., 2016), navigation and teleoperation (Dias et al., 2015; Papachristos and Alexis, 2016; Yew et al., 2017), socially assistive robots (Čaić et al., 2020), and human-robot collaboration (Gurevich et al., 2015; Walker et al., 2018; Makhataeva et al., 2019; Wang and Rau, 2019). AR has also revolutionized the concepts of human-robot interaction (HRI) by providing a user-friendly medium for perception, interaction, and information exchange (De Tommaso et al., 2012).

What has preceded affirms that the benefits of combining AI and AR in robotics are manifold, and special attention should be given to such efforts. There are several review papers highlighting the integration of augmented reality to robotics from different perspectives such as human-robot interaction (Green et al., 2008; Williams et al., 2018), industrial robotics (De Pace et al., 2020), robotic-assisted surgery (L. Qian et al., 2020), and others (Makhataeva and Varol, 2020). Similarly, there exist papers addressing the potential of integrating artificial intelligence in robotics as reviewed in Loh (2018), De Pace et al. (2020) and Tussyadiah (2020). A recent review (Makhataeva and Varol, 2020) summarizes the work done at the intersection of AR and Robotics, yet it only mentions how augmented reality has been used within the context of robotics and does not touch on the intelligence in the system from different perspectives as highlighted in this paper. Similarly, another systematic review (Norouzi et al., 2019) presented the convergence of three technologies: Augmented reality, intelligent virtual agents, and internet of things (IOT). However, it did not focus on robotics as the main intelligent systems and even excludes agents having physical manifestations of humanoid robots. Consequently, this paper systematically reviews literature done over the past 10 years at the intersection of AI, AR, and robotics. The purpose of this review is to compile what has been previously done, analyze how augmented reality is supporting the integration of artificial intelligence in robotics and vice versa, and suggest prospective research opportunities. Ultimately, we contribute to future research through building a foundation on the current state of AR and AI in robotics, specifically addressing the following research questions:

1) What is the current state of the field on research incorporating both AR and AI in Robotics?
2) What are the various elements and disciplines of AR and AI used and how are they intertwined?
3) What are some of the current applications that have benefited from the inclusion of AR and AI? And how were these applications affected?

To the best of our knowledge, this is the first literature review combining AR and AI in robotics where papers are systematically collected, reviewed, and analyzed. A categorical analysis is presented, where papers are classified based on which technology supports the other, i.e., AR supporting AI or vice versa, all under the hood of robotics. We also classify papers into their perspective robotic applications (for example grasping) and explain how this application was improved. Research questions 1 and 2 are answered in *Results*, and research question 3 is answered in *Discussion*.

The remainder of the paper is organized according to the following sections: Methods, which specifies the survey methodology adopted as well as inclusion and exclusion criteria, Results, which presents descriptive statistics and analysis on the total number of selected papers in this review (29 papers), Discussion, which presents an analysis on each paper from different perspectives, and finally Concluding Remarks, which highlights key findings and proposes future research.

## Methods

**FIGURE 1:**
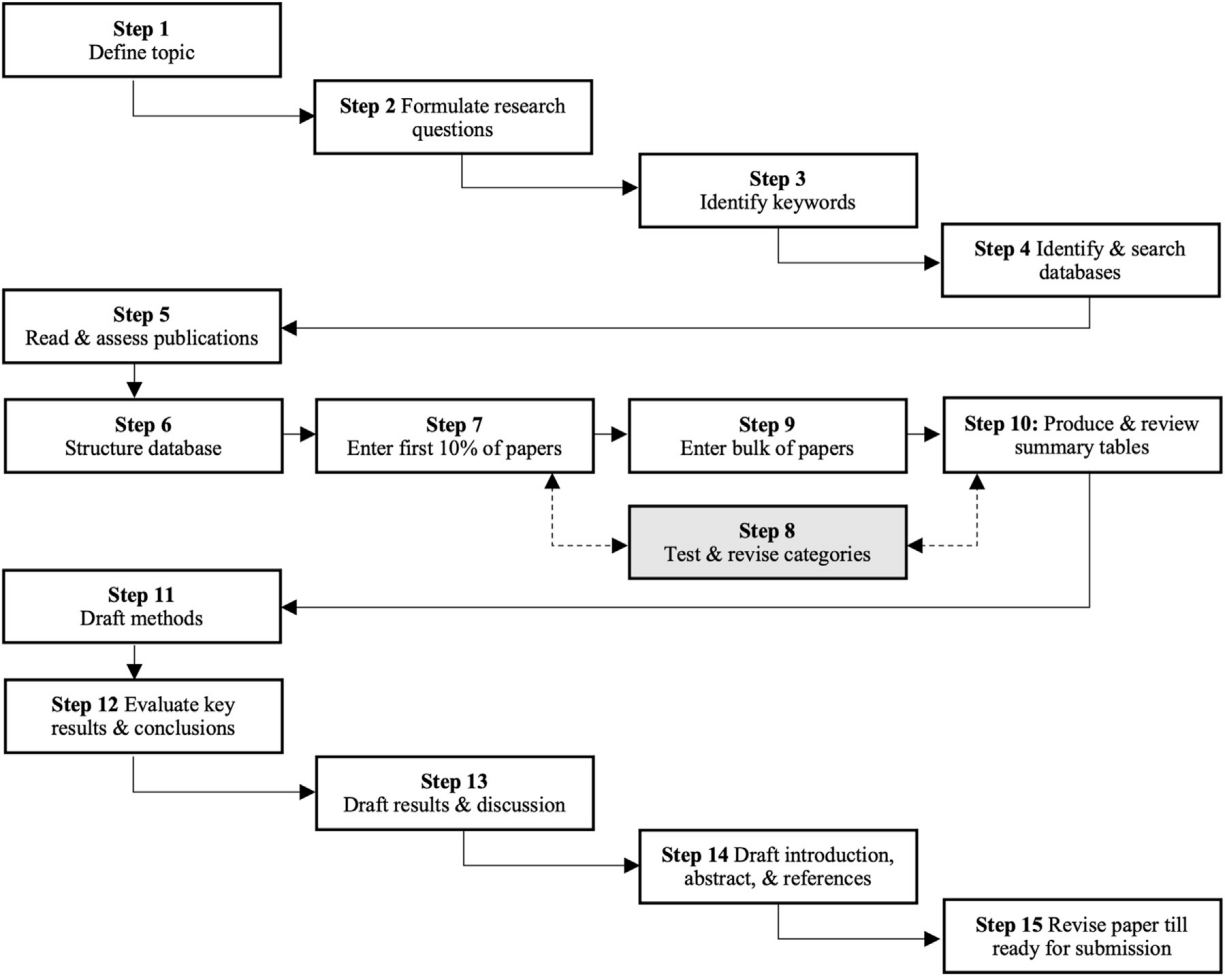
The adopted systematic approach in this review paper.

This paper follows a systematic approach in collecting literature. We adopt the systematic approach set forth in Pickering and Byrne (2014), which is composed of 15 steps as illustrated in Figure 1.

Steps 1 and 2 were explicitly identified in the *Introduction*. This section outlines the used keywords (step 3) and the used databases (step 4).

### Search Strategy and Data Sources

Regarding keywords, this review targets papers that combine augmented reality with artificial intelligence in robotics. The first source used was Google Scholar denoted by G. Initially, we excluded the words surgery and education (search keys G1, G2, and G3) to narrow down the total number of output papers. Concurrently, there are several papers reviewing AI Robots in surgical applications (Loh, 2018; Andras et al., 2020; Bhandari et al., 2020) and AI in education (Azhar et al., 2020; Chen et al., 2020a; Chen et al., 2020b). Then, search keys G4 and G5 were used (Where we re-included the terms “surgery” and “education”) to cover a wider angle and returned a large number of papers, upon which we scrutinized only the first 35 pages. The second source of information is Scopus Database denoted by S, upon which two search keys were used, S1 and S2, and the third is ICRA 2020 proceedings. Finally, the references and citations of the corresponding selected outputs from these three sources were checked.

The time range of this review includes papers spanning the years between 2010 and 2020. Note that the process of paper collection for search keys G1, G2, G3, G4, S1, and S2 started on the 30^th^ of June and ended in July 21^st^ 2020. G5 search key was explored between August 11^th^ and August 20^th^, 2020 and finally, ICRA 2020 proceedings were explored between August 20^th^ and August 31^st^ 2020.

### Study Selection Criteria

The selection process was as follows: First duplicates, patents, and non-English papers were excluded. Then, some papers were directly excluded by scanning their titles, while others were further evaluated by looking into their abstract and keywords and downloading those that are relevant. Downloaded papers are then scanned through quickly going over their headers, sub-headers, figures, and conclusions. Starting from a total of 1,200, 329, and 1,483 papers from Google Scholar, Scopus database, and ICRA proceedings respectively, the total number of selected papers were funneled down to 13, 8, and 3 papers, respectively. After that, we looked into the references and citations of these 24 papers and selected a total of five papers. The inclusion and exclusion criteria were as follows:

### Exclusion Criteria

• Papers with a non-English content
• Duplicate papers
• Patents

### Inclusion Criteria

• The application should directly involve a robot
• Artificial Intelligence is involved in the Robotics Application. Although the words artificial intelligence and machine learning are used interchangeably in this paper, most of the cited work is more accurately a machine learning application. Artificial intelligence remains the broader concept of machines acting with intelligence and thinking as humans, with machine learning being the subset of algorithms mainly concerned with developing models based on data in order to identify patterns and make decisions.
• An Augmented Reality technology is utilized in the paper.

**FIGURE 2:**
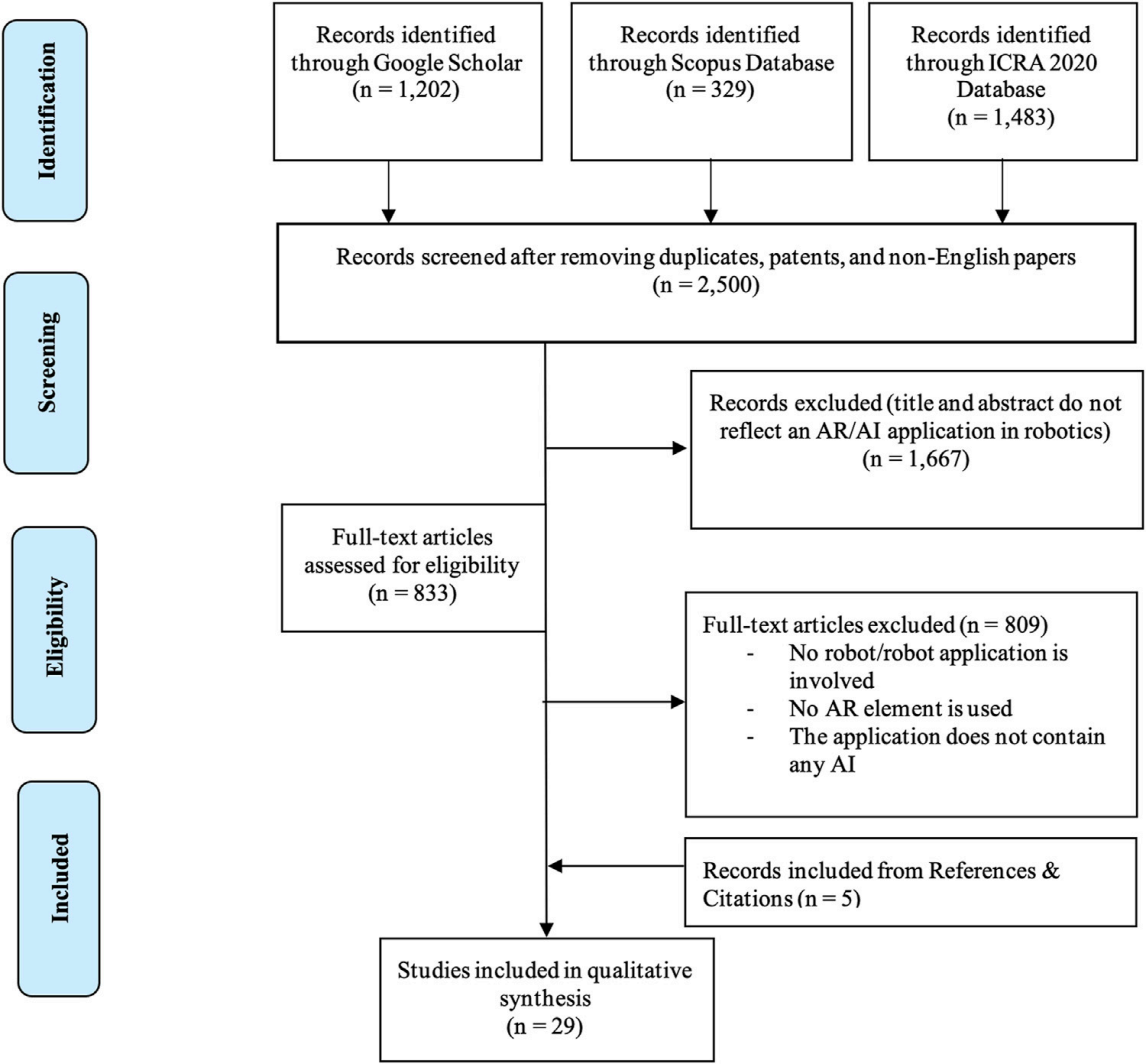
The Preferred Reporting Items for Systematic Reviews and Meta-Analyses (PRISMA) chart.

The process flow is also illustrated in Figure 2 according to the Preferred Reporting Items for Systematic Reviews and Meta-Analyses (PRISMA) guidelines (Moher et al., 2009).

## Results

A total of 29 papers were selected and individually examined through checking their abstracts, conclusions, and analyzing their main content. This section presents how the collected literature was classified into categories and presents some descriptive statistics visualized through figures and tables.

### Categorization

There are two parallel categorizations in this paper: a theme-based categorization and an application-based categorization. Initially, all papers were grouped into two clusters based on a theme-based grouping of how AR and AI serve each other in a certain robotics application. The two distinguished clusters were as follows: AR supports AI, and AI supports AR. Each of these clusters is further explained below along with the total number of papers per group.

### AR Supports AI (18 Papers)

This cluster groups papers in which a certain augmented reality visualization facilitates the integration of artificial intelligence in robotics. An example is an augmented reality application which provides visual feedback that aids in AI robot performance testing.

### AI Supports AR (11 Papers)

Papers in which the output of AI algorithms and neural networks support an accurate display of augmented reality markers and visualizations.

Another remarkable pattern was noted among the 29 papers in terms of the specific robotics application that this AR-AI alliance is serving. In consequence, a parallel categorization of the 29 reviewed articles is realized, and three clusters were distinguished as follows:

### Learning (12 Papers)

A robot learns to achieve a certain task, and the task is visualized to the human using AR. This category combines papers on learning from demonstration (LFD) and learning to augment human performance.

### Planning (8 Papers)

A robot intelligently plans a certain path, task, or grasp, and the user can visualize robot information and feedback through AR.

### Perception (9 Papers)

A robot depends on AI vision algorithms to localize itself or uses object detection and recognition to perceive the environment. AR serves here in identifying the robot’s intent.

### Statistical Data

**FIGURE 3:**
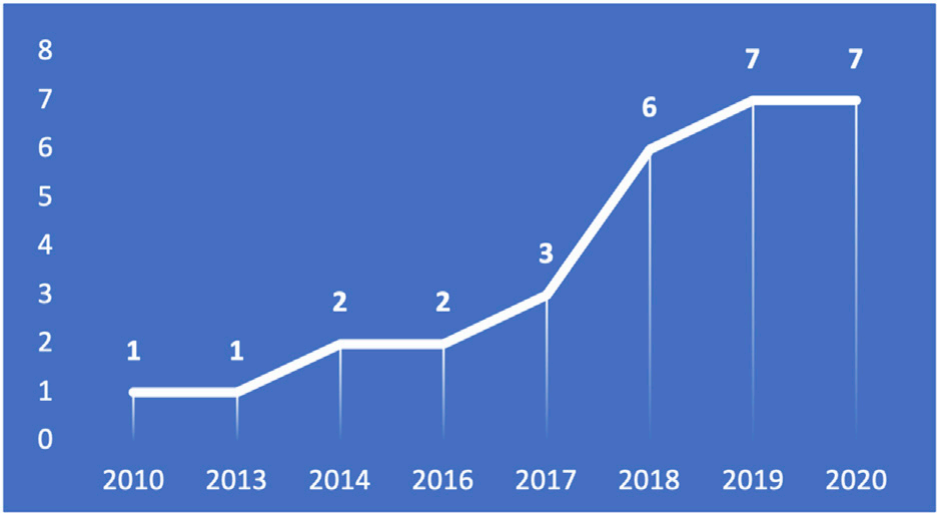
The growing rate of published papers addressing our target topic over time.

**FIGURE 4:**
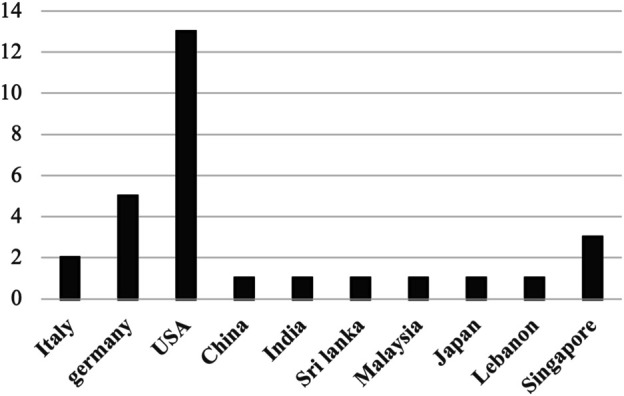
The distribution of reviewed papers over their countries of origin.

For the sake of analyzing historical and graphical aspects of the reviewed topic, Figures 3, 4 present the yearly and regional distribution of reviewed papers, respectively. Historically, the number of publications integrating AR and AI in robotics applications has increased significantly between the years 2010 and 2020 (2020 is equal to 2019 but the year has not ended yet), demonstrating the growing interest in combining the capabilities of AR and AI to solve many challenges in robotics applications. Regionally, the united states is the leading country in terms of the number of published articles, followed by Germany. Note that we only considered the country of the first author for each paper.

Additional quantitative data are detailed in Table 1. For each article, the table identifies five types of information: The AR technology and platform, the type of robot platform, the used AI algorithm, and to which cluster (from each category) it belongs. Overall, the most commonly used AR component is the HMD (48% of papers), mainly Microsoft HoloLens (Microsoft HoloLens, 2020), Oculus Rift (Oculus, 2021), or custom designed headsets. This is followed by desktop-based monitors (28%) and AR applications on handheld tablets and mobile phones (21%). Projection-based spatial AR were the least implemented (3%), which can be explained by the added complexity of the setup and lack of mobility. Unity3D game engine was the most commonly used for developing AR applications and visualizations, in comparison to Unreal Engine. Other options were using the Tango AR features supported by the Google Tango tablet or creating applications from scratch using the OpenGL graphics library. Regarding the type of robot used, aerial robots, such as UAVs and drones, were the least utilized (13%) in comparison to mobile robots (48%) and robotic arms (39%). Deep Neural networks were the most investigated in literature (52%) along with other state-of-the-art machine learning algorithms. Furthermore, the majority of papers were involved in creating visualizations that support AI integration to robotics, rather than implementing AI to enhance the augmented reality application in robotics.

**TABLE 1:** Descriptive elements on the type of the used AR Component, robotics platform, AI component, and categorization for all reviewed papers.

References	Tablet/Desktop	AR components						Robot				AI components												Category 1		Category 2		
		HMD	Spatial	Unreal	Unity	OpenGL	Tango	Mobile	Arm	Aerial	ADIOS	Neural networks	FP-Growth	Q- learning	SVM	SVD	KNN	Regression	MDP	Expectation maximization	DBSCAN	DTW	Gradient boosting	AR supports AI	AI supports AR	Learning	Planning	Perception
Ong et al., (2010)	—	x	—	—	—	x	—	—	x	—	—	x	—	—	—	—	—	—	—	—	—	—	—	x	—	x	—	—
Fang et al., (2013)	x	—	—	—	—	x	—	—	x	—	—	x	—	—	—	—	—	—	—	—	—	—	—	x	—	x	—	—
Fang et al., (2014)	x	—	—	—	—	x	—	—	x	—	—	x	—	—	—	—	—	—	—	—	—	—	—	x	—	x	—	—
Ghiringhelli et al., (2014)	x	—	—	—	—	—	—	x	—	—	—	—	—	—	—	—	—	x	—	—	—	—	—	—	x	—	—	x
Measurable Augmented Reality for Prototyping Cyberphysical Systems (2016)	—	—	x	—	—	x	—	x	—	x	—	—	—	—	—	—	—	—	x	—	—	—	—	x	—	—	x	—
Sawarkar et al., (2016)	—	x	—	—	—	—	—	x	—	x	—	x	—	—	—	—	—	—	—	—	—	—	—	—	x	—	—	x
Muvva et al. (2017)	—	x	—	—	x	—	—	x	—	—	—	—	—	x	—	—	—	—	—	—	—	—	—	x	—	—	x	—
Chakraborti et al., (2017)	—	x	—	—	—	—	—	—	x	—	—	—	—	x	x	—	—	—	—	—	—	—	—	x	—	—	x	—
Warrier and Devasia, (2018)	—	x	—	—	—	—	—	—	x	—	—	—	—	—	—	—	—	x	—	—	—	—	—	—	x	x	—	—
Wang et al., (2018)	x	—	—	—	—	x	—	x	—	—	—	x	—	—	—	—	—	—	—	—	—	—	—	—	x	—	—	x
Gradmann et al., (2018)	x	—	—	—	—	—	x	—	x	—	—	—	—	—	—	—	—	—	—	—	x	—	—	x	—	x	—	—
Sprute et al., (2019b)	x	—	—	—	—	—	x	x	—	—	—	x	x	—	—	—	—	—	—	—	x	—	—	x	—	x	—	—
Bentz et al., (2019)	—	X	—	—	—	—	—	—	—	x	—	—	—	—	—	—	—	—	—	x	—	—	—	—	x	x	—	—
Corotan and Irgen-Gioro, (2019)	X	—	—	—	—	x	—	x	—	—	—	—	—	x	—	—	—	—	—	—	—	—	—	x	—	—	x	—
Puljiz et al., (2019)	—	X	—	—	X	—	—	—	x	—	—	x	—	—	—	—	—	—	—	—	—	—	—	—	x	—	—	X
Liu et al., (2018)	—	X	—	—	X	—	—	—	x	—	x	—	—	—	—	—	—	—	—	—	—	—	—	x	—	x	—	—
(Tay et al., n.d.)	X	—	—	—	X	—	—	X	—	—	—	—	—	—	—	—	—	—	—	—	—	—	x	—	x	x	—	—
De Gregorio et al., (2020)	X	—	—	—	—	—	—	—	X	—	—	x	—	—	—	—	—	—	—	—	—	—	—	x	—	—	—	X
Kästner et al. (2020)	—	X	—	X	—	—	—	X	—	—	—	x	—	—	—	—	—	—	—	—	—	—	—	—	x	—	—	X
Dias et al., (2020)	X	—	—	—	—	—	—	X	—	—	—	x	—	—	—	—	—	—	x	—	—	—	—	x	—	x	—	—
Weisz et al., (2017)	X	—	—	—	—	—	—	—	X	—	—		—	—	—	—	x	—	—	—	—	—	—	x	—	—	x	—
Hastie et al., (2018)	X	—	—	—	—	—	—	X	—	—	—	x	—	—	—	—	—	—	—	—	—	—	—	x	—	—	x	—
Cao et al., (2019)	—	X	—	—	X	—	—	—	X	—	—		—	—	—	—	—	—	—	—	—	X	—	X	—	x	—	—
Kastner et al., (2020)	—	X	—	—	X	—	—	X	—	—	—	x	—	—	—	—	—	—	—	—	—	—	—	—	x	—	—	X
Zhang et al., (2020)	X	—	—	—	X	—	—	X	—	—	—	x	—	—	—	—	x	—	—	—	—	—	—	—	x	—	—	X
Zein et al., (2020)	X	—	—	—	—	—	—	—	—	X	—		—	—	X	—	—	—	—	—	—	—	—	X	—	—	x	—
El Hafi et al., (2020)	—	X	—	—	X	—	—	X	—	—	—	x	—	—	—	—	—	—	—	—	—	—	—	—x	—	—	x	—
(Chu et al., 2018.)	—	X	—	—	X	—	—	—	X	—	—	x	—	—	—	—	—	—	—	—	—	—	—	—	x	—	—	X
Gadre, (2018)	—	X	—	—	X	—	—	—	x	—	—	—	—	—	—	—	—	x	X	—	—	—	—	x	—	x	—	—

**FIGURE 5:**
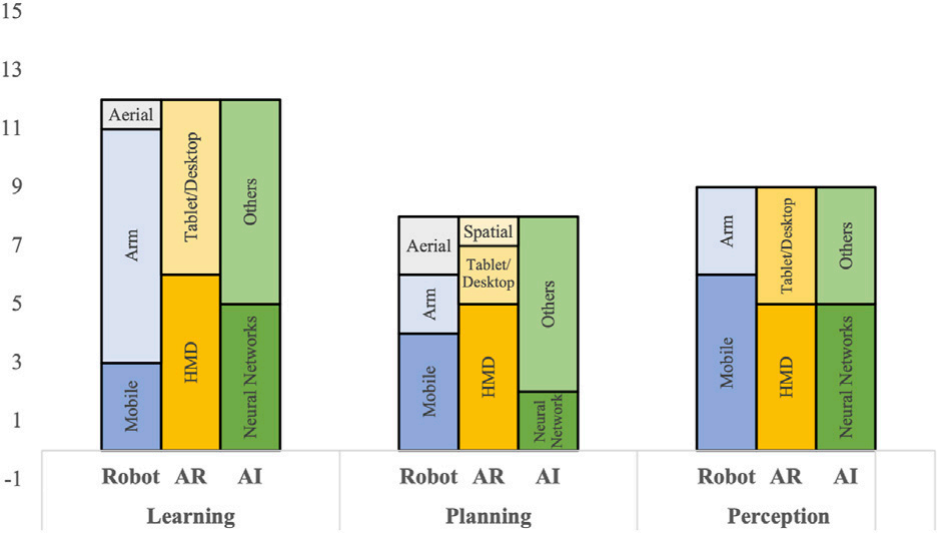
The quantity distribution of three factors: Robot platform, AR technology, and AI method, over the three robot applications: Learning, Planning, and Perception.

Another set of distinctive features were extracted through analyzing three attributes, mainly the type of robot platform used, the type of AR technology employed, and the nature of the AI method performed, for each of the three robotics applications. The results are depicted in Figure 5. The majority of papers (around 70%) that fall under the “Learning category” were using robot arms and manipulators as their robot platform. This is mainly because the Learning category reviews the learning from demonstration application, which is historically more common for industrial robotics applications in which a user demonstrates the trajectory of the end effector (EE) of a robot arm (Billard et al., 2008; Mylonas et al., 2013; Zhu and Hu, 2018) than in the context of mobile robots (Simões et al., 2020) or aerial robots (Benbihi et al., 2019). On the other hand, around 70% of reviewed papers targeting robot “Perception” applications were using mobile robots. The reason is that vision-based localization algorithms are usually more ubiquitous for mobile robots (Bonin-Font et al., 2008) compared to the other two platforms. The three robot platforms were almost equally distributed in the “Planning” category with a relatively higher prevalence of mobile robots.

Regarding the type of AR hardware/technology used, it was noted that the HMD was the most commonly used for all robotics applications covered, followed by the tablet or the desktop-based monitor. Spatial AR, or projection-based AR, was the least commonly used given its rigidness in terms of mobility and setup. As for the used AI, there was a variety of methods used, including regression, support vector machine (SVM), and Q-learning. However, neural networks, including YOLO and SSD deep neural networks, were the more commonly used across the three robotics applications. Neural networks were utilized in 42, 25, and 80% of the reviewed papers in the learning, planning, and perception categories, respectively.

## Discussion

Augmented reality technology has created a new paradigm for human-robot interaction. Through enabling a human-friendly visualization of how a robot is perceiving its environment, an improved human-in-the-loop model can be achieved (Sidaoui et al., 2019; Gong et al., 2017). The use of AR technology for robotics has been elevated by the aid of several tools, mainly Vuforia Engine (Patel et al., 2019; Makita et al., 2021; Comes et al., 2021), RosSharp (Kästner and Lambrecht, 2019; Rosen et al., 2019; Qiu et al., 2021), ARCore (Zhang et al., 2019; Chacko et al., 2020; Mallik and Kapila, 2020), and ARKit (Feigl et al., 2020; McHenry et al., 2021). ARCore and ARKit are tools that have enhanced the AR experience for motion tracking, environmental understanding, light estimation, among other features. RosSharp has provided an open-source software for communication between ROS and Unity, which have greatly facilitated the use of AR for robot applications and provided useful easy-access functionalities, such as publishing and subscribing to topics and transferring URDF files.

In this section, 29 papers are analyzed in two parallel categorizations as explained in *Results*, a *theme-based analysis* capturing the relation between AR and AI in a robotic context (AR supports AI, AI supports AR) and an *application-based analysis* focusing on the perspective of how the robotic application itself was improved. We have also compiled a qualitative table (Table 2) highlighting several important aspects in each paper. The highlighted aspects include the type of robot used, the nature of the experiment and number of human subjects, the human-robot interaction aspect, and the advantages, disadvantages and limitations of integrating AR and AI.

**TABLE 2:** Qualitative information and analysis of each paper.

References	Robot		Methodology		Human-robot interaction
	Real/Virtual	Type	Human subject	Experiment	
Ong et al. (2010)	Virtual	PUMA 560 industrial robot	In-house author case study	The user provides several demonstrations for a system to learn an unknow curve	Demonstrating robot paths instead of explicitly programming them
Fang et al. (2013)	Real	SCORBOT-ER VII manipulator	11 Participants	Participants were asked to plan the EE orientation along some visible curve. Also they filled a questionnaire	Making robot path planning easier for non-technical users
Fang et al. (2014)	Real	SCORBOT-ER VII manipulator	12 Participants	Participants performed two case studies, including a pick and place task	Making robot path planning easier for non-technical users
Ghiringhelli et al. (2014)	Real	Swarm Robots	In-house author case study	Robot tracking and visualization modules were implemented and tested	Simplifying the debugging of swarm robots for human developers
Measurable Augmented Reality for Prototyping Cyberphysical Systems, (2016)	Both	Autonomous Aerial Vehicle	In-house author case study	Given a set of randomly generated destinations, a swarm of quadrotors plans paths while avoiding self and human collisions	Seeking a collision free human-robot shared workspace
Sawarkar et al. (2016)	Real	UGV	In-house author case study	Controlling the pan-tilt of an IP camera through a HMD and identifying the terrorist probabilities of two cases	Making teleoperation and monitoring of robots intuitive
Muvva et al. (2017)	Real	Arduino-controlled mobile robot	In-house author case study	Reaching a predetermined destination while avoiding physical and augmented objects	-Implementing a cost-effective solution for training a robot in RL.
Chakraborti et al. (2017)	—	—	—	Theoretical: A framework for an augmented workspace in HRI.	Solving the impedance mispatch in HRI.
Warrier and Devasia, (2018)	Real	Kinova Robot	In-house author case study	Tracking the operator’s motion while visualizing the output trajectory and desired one	Demonstrating the robot trajectory through tracing human motion
Wang et al. (2018)	Real	UGV	In-house author case study	Detecting a target in the Robot’s environment	Displaying the marker on the human’s remote monitoring device
Gradmann et al. (2018)	Real	KUKA Lightweight Robots	In-house author case study	Pick and place of objects clustered using DBSCAN.	Teleoperating the robot in AR.
Sprute et al. (2019a)	Real	Mobile Robots	15 Participants	Users were asked to define virtual borders via an AR Google Tango tablet.	Restricting robot workspace in HR shared environments
Bentz et al. (2019)	Real	Asctec Hummingbird aerial robot	10 Participants	Students were asked to perform a multitasking scenario assisted with an aerial robot	Developing a collaborative human-robot multitasking framework
Corotan and Irgen-Gioro, (2019)	Real	Arduino-controlled mobile robot	In-house author case study	A robot navigating a floor to reach a goal destination while relying only on ARCore capabilities	- Developing an AR-based indoor navigation system
Puljiz et al. (2019)	Real	KUKA KR-5 robot	In-house author case study	Testing the performance of two referencing algorithms	Making the AR setup experience less tedious
Liu et al. (2018)	Real	Baxter Robot	In-house author case study	Teaching a robot to open various bottles	Patching new skills to a robot in real time through an intuitive interphase
Tay et al. (n.d.)	Real	Turtlebot 3 Waffle	In-house author case study	Collecting IMU data during robot navigation and testing performance of two algorithms in real time	Simplifying the process of debugging
De Gregorio et al. (2020)	Real	Robot Manipulator	In-house author case study	Creating two novel datasets for industrial robotic applications using the ARS pipeline	Projecting representations on items through a user-friendly AR interphase
Kästner et al. (2020)	Real	Kuka Mobile Youbot	In-house author case study	Evaluating the performance of four algorithms in the 6D pose detection of the mobile robot	Making the AR setup experience less tedious
Dias et al. (2020)	Real	Mobile robots and a robotic arm	50 Participants	Participants were asked to choose a sequence of item placements for 11 task variants to be used as a training dataset.	Teaching robots new task variants through providing AR enabled demonstrations
Weisz et al. (2017)	Real	Industrial Stäubli TX60L Robotic arm and a BarrettHand gripper	Five healthy subjects and one impaired user	Several grasping experiments	Supporting impaired individuals through an assistive grasping system
Hastie et al. (2018)	Real	Autonomous Underwater Vehicle	—	Implementing the ORCA interphase to provide explanations on robot perception and planning algorithms	Visualizing robot intent through increasing its transparency
Cao et al. (2019)	Real	Mobile robot with Arduino Braccio Arm	12 Participants	Users were asked to perform continuous motion, define ghosts, and complete an assembly task. Also, they filled a questionnaire	A framework for human-robot collaborative task
Kastner et al. (2020)	Real	Kuka Mobile Youbot	In-house author case study	Testing a modified version of the VoteNet architecture	Making the AR setup experience less tedious
Zhang et al. (2020)	Real	ABB Yumi robot	In-house author case study	Grasping of different objects detecting using the developed pipeline	Visualizing robot intent in terms of the planned grasps
Zein et al. (2020)	Virtual	Parrot AR Drone	In-house author case study	Drone autonomously navigating a path in a simulated Gazebo environment	A framework to assist users in UAV teleoperation
El Hafi et al. (2020)	Real	Toyota Human Support Robot	In-house author case study	Customer interaction task	Achieving human-robot integration in customer-service scenarios
Chu et al. (2008)	Real	7 DOF Robotics arm	In-house author case study	Pick and place task using the TDS input	A human-robot collaborative framework for people with disabilities
Gadre, (2018)	Real	Baxter Robot	In-house author case study	Pick and place tasks for several objects	Creating an intuitive AR interphase for collecting demonstration

### Theme-Based Analysis

The two themes highlighted here depend on the nature of the AR-AI alliance. Consequently, 18 papers in which an augmented reality technology is facilitating the integration of AI to robotics are reviewed under the “AR supports AI” theme, and 11 papers in which AI has been integrated to enhance the AR experience for a certain robotics application are reviewed under the “AI supports AR” theme.

#### AR Supports AI

In this cluster, augmented reality is used as an interface to facilitate AI, such as visualizing the output of AI algorithms in real-time. Papers are grouped depending on the type of robotic platform used: mobile robots, robotic arms, or aerial robots. Some papers contain both and are categorized based on the more relevant type.

### Mobile Robots

An AR interface was developed in El Hafi et al. (2020) for an intelligent robotic system to improve the interaction of service robots with non-technical employees and customers in a retail store. The robot performs unsupervised learning to autonomously form multimodal place categorization from a user’s language command inputs and associates them to spatial concepts. The interface provided by an HMD enables the employee to monitor the robot’s training in real-time and confirm its AI status.

After investigating possible interfaces that allow user-friendly interactive teaching of a robot’s virtual borders (Sprute et al., 2019a), the authors in Sprute et al. (2019b) used a Google-Tango tablet to develop an AR application which prompts the user to specify virtual points on a live video of the environment from the tablet’s camera. The used system incorporates a Learning and Support Module which learns from previous user-interactions and supports users through recommending new virtual borders. The borders will be augmented on the live stream and the user can directly select and integrate them to the Occupancy Grid Map (OGM).

An augmented reality framework was proposed in Muvva et al. (2017) to provide a cost-effective medium for training a robot an optimal policy using Q-learning. The authors used ODG-R7 glasses to augment virtual objects at locations specified by fiducial markers. A CMU pixy sensor was used to detect both physical and virtual objects.

An AR mobile application was developed in Tay et al. (n.d.) that can inform the user of specific motion abnormalities of a Turtlebot, predict their causes, and indicate future failure. This information will be augmented on the live video of a mobile phone and sent to the user via email. The system uses the robot’s IMU data to train a gradient boosting algorithm which classifies the state of the motor into fault conditions indicating the level of balancing of the robot (tilting). This system decreases the downtime of the robot and the time spent on troubleshooting.

The authors in Corotan and Irgen-Gioro (2019) investigated the capabilities of augmented reality (ARCore) as an all in one solution for localization, indoor routing, and detecting obstacles. The application runs on a Google Pixel smartphone, which acts as both the controller (through a three-view user interface) and the sensor. Using its on-board localization features, an optimal path is planned from a starting position to an end position based on a Q-learning algorithm.

Omidshafiei et al. (Measurable Augmented Reality for Prototyping Cyberphysical Systems, 2016) implemented an AR environment that provides visual feedback of hidden information to assist users in hardware prototyping and testing of learning and planning algorithms. In this framework, a ceiling-mounted projection system augments the physical environment in the laboratory with specific mission-related features, such as visualizing the state observation probabilities. In this system, the tracking of mobile and aerial robots is based on motion-capture cameras. Similarly, Hastie et al. (2018) presented the MIRIAM interface developed by the ORCA Hub: a user-centered interface that supports on-demand explainable AI through natural language processing and AR visualizations.

### Robotic Arms

An Android mobile AR application was developed in Dias et al. (2020) as a training interface for a multi-robot system to perform a task variant. The tablet acts as a data collection interface based on the captured input demonstrations of several users. The application visualizes detected robots (using AR markers) and enables each user to construct a toy building of their choice through sequential tasks. Deep Q-learning (Hester et al., 2017) has been employed to learn from the sequence of user demonstrations, predict valid variants for the given complex task, and achieve this task through a team of robots. The accuracy achieved in task prediction was around 80%.

The authors in Warrier and Devasia (2018) implemented a *Complex Gaussian Process Regression* model to learn the intent of a novice user during his/her teaching of the End Effector (EE) position trajectory. A Kinect camera captures the user’s motion, and an AR HMD visualizes the desired trajectory versus the demonstrated trajectory, which allows the operator to estimate the error (i.e., difference between the two trajectories) and correct accordingly. This approach was tested by a single operator and showed a 20% decrease in the tracking errors of demonstrations compared to manual tracking.

AR solutions were investigated in Ong et al. (2010) to correct for model mismatches in programming by demonstration (PBD), where they used an HMD as a feedback and data collection interface for robot path planning in an unknown environment. The user moves a virtual robot (a probe with AR markers) along a desired 3D curve with a consistent orientation, while evaluating the drawn curve using AR. The collected data points are then fed to a three-stage curve learning method, which increased the accuracy of the desired curve. The system was further enhanced in Fang et al. (2013) through considering robot dynamics, basically the end effector (EE) orientation. Once the output curve is generated, a collision-free volume (CFV) is displayed and augmented on a desktop screen to the user who can select control points for EE orientation. Some limitations in the proposed interface were found, such as the difficulty in aligning the virtual robot with the interactive tool, occluding markers or moving them out of the camera’s view, and selecting inclination angles that are not within range, causing the EE to disappear from the display. Consequently, the used AR visual cues were further developed for a robust HRI in Fang et al. (2014), such as the use of virtual cones to define the orientation range of the EE, colors to distinguish dataset points, control points, and points outside the range of the CFV, and an augmented path rendered by a set of selected control points.

A HoloLens HMD was also used in Liu et al. (2018) as an AR interface in the teaching process of interpretable knowledge to a 7-DoF Baxter robot. The full tree of robot coordinates TF and latent force data were augmented on the physical robot. The display also offers the user to turn on the robot’s learned knowledge represented by a “Temporal And-Or graph,” which presents live feedback of the current knowledge and the future states of the robot.

A semi-automatic object labeling method was developed in De Gregorio et al. (2020) based on an AR pen and a 2D tracking camera system mounted on the arm. In this method, a user first outlines objects with virtual boxes using an AR pen (covered with markers) and a robot acquires different camera poses through scanning the environment. These images are used to augment bounding boxes on a GUI which enables the user to refine them.

The authors in Gadre (2018) implemented a training interface facilitated by Microsoft HoloLens for learning from demonstration. The user can control the EE position by clicking commands on a transparent sphere augmented on the EE and use voice commands to start and end the recording of the demonstration. Through clicking on the sphere at a specific EE position, the system will store it as a critical point (CP) and augment a transparent hologram of the robot on its position as a visual reminder of all saved CPs. The saved CPs are then used to learn a Dynamic Movement Primitive (DMV).

A spatial programming by demonstration (PBD) called GhostAR was developed in Cao et al. (2019), which captures the real-time motion of the human, feeds it to a dynamic time warping (DTW) algorithm which maps it to an authored human motion, and outputs corresponding robot actions in a human-lead robot-assist scenario. The captured human motions and the corresponding robot actions are saved and visualized to the user who can observe the complete demonstration with saved AR ghosts of both the human and robot and interactively perform edits on robot actions to clarify user intent.

The authors in Zhang et al. (2020) created the Dex-Net deep grasp planner, a distributed open-source pipeline that can predict 100 potential grasps from the object’s depth image based on a pre-trained Grasp Quality CNN. The grasp with the highest Quality value will be overlaid on the object’s depth map and visualized on the object through an AR application interface provided by ARKit. The system was able to produce optimal grasps in cases where the top-down approach doesn’t detect the object’s complex geometry.

An AR assistive-grasping system was implemented in Weisz et al. (2017) that can be used by impaired individuals in cluttered scenes. The system is facilitated by a surface electromyography (sEMG) input device (a facial muscle signal) and can be evaluated using an augmented reality desktop-based display of the grasping process. The interface allows a visualization of the planned grasp. The probabilistic road map planner (Kavraki et al., 1996) was used to verify the reachability of an object and a K-nearest neighbor (KNN) classifier for classifying objects into reachable and unreachable.

The authors in Chakraborti et al. (2017) proposed combining AR technology with electroencephalographic (EEG) signals to enhance Human-robot collaboration specifically in shared workspaces. Two AR interaction modalities were implemented via an HMD. The first facilitates the human-in-the-loop task planning while the other enhances situational awareness. Through observing the emotions from EEG signals, the robot can be trained through reinforcement learning to understand the user’s preferences and learn the process of human-aware task planning.

### Aerial Robots

A teleoperation system was developed in Zein et al. (2020) that recognizes specific desired motions from the user joystick input and accordingly suggests to auto-complete the predicted motion through an augmented user interface. The proposed system was tested on Gazebo using a simulated Parrot Ar. Drone 2.0 and performed better than manual steering by 14.8, 16.4, and 7.7% for the average distance, time, and Hausdorff metric, respectively.

The authors in Bentz et al. (2019) implemented a system in which an aerial collaborative robot feeds the data from the head motions of a human performing a multitasking job to an Expectation-Maximization that learns which environment views have the highest visual interest to the user. Consequently, the co-robot is directed to capture these relevant views through its camera, and an AR HMD supplements the human’s field of view with views when needed.

Overall, the advantages of augmented reality in facilitating the integration of AI to robotics applications are manifold. AR technologies can provide a user-friendly and intuitive medium to visualize the learning process and provide the live learned state of the robot. They also provide a medium for the robot to share its present and future intent, such as the robot perceived knowledge and the robot’s planned actions based on its AI algorithms. Although the AR HMDs - such as those provided by Microsoft HoloLens and Oculus Rift - are the most commonly used for an intuitive HRI, they still have their limitations such as their narrow field of view (FOV) and impractical weight. Other AR interfaces used included mobile phones, tablets, and desktop displays. The latter is more practical in simulations, otherwise, the user will need to split attention between the actual robot and the augmented display. Tablets and mobile phones are generally more intuitive but impractical in situations where the user has to use both hands. Spatial AR, also known as projection-based AR, is less used due to its mobility restrictions.

#### AI Supports AR

In this cluster, AI contributes to an accurate and more reliable augmented reality application, or interface, such as applying deep learning for detecting obstacles in the robot’s path. Papers are also grouped depending on the type of robotic platform used.

### Mobile Robots

The authors in Ghiringhelli et al. (2014) implemented an AR overlay on the camera view of a multi-robot system. The system supports three types of information: textual, symbolic, and spatially situated. While the first two reveal insights about the internal state of each robot without considering its orientation or camera perspective, spatially situated information depends on how the robot perceives its surrounding environment and are augmented on each robot using its frame of reference. Properly augmenting information depends on a visual tracking algorithm that identifies robots from the blinking code of an onboard RGB LED.

In Wang et al. (2018), the authors used deep learning to obtain the location of a target in the robot’s view. The robot first runs simultaneous localization and mapping (SLAM) to localize and map the place in an urban search and rescue scenario. Once the robot detects a target in the area, an AR marker is placed on its global coordinate and displayed to the user on the augmented remote screen. Even when the detected target is not within display, the location of the marker changes according to its place relative to the robot.

The authors in Kastner et al. (2020) developed a markerless calibration method between a HoloLens HMD and a mobile robot. The point cloud data acquired from the 3D depth sensor of the AR device are fed into a modified neural network based on VoteNet. Although the approach was feasible in terms of an accurate localization and augmentation of the robot by a 3D bounding box, the intensive live processing operations of point cloud data was very slow. Two seconds was the time needed for the user to stay still while the neural network processes the incoming data, which can be impractical and lead to a bad user experience.

Alternatively, Kästner et al. (2020) investigated using the 2D RGB data provided by the HoloLens instead, which is relatively faster to process than 3D data and can be applied to any AR device. SSPE neural networks were deployed in order to localize the six DOF pose of a robot. Meanwhile, the resulting bounding boxes are augmented to the user, who can evaluate the live training process. This method is around 3% less accurate than the first one but almost 97% faster.

### Robotic Arms

The authors in Puljiz et al. (2019) reviewed the referencing and object detection methods used in the robotics field in general and the referencing methods currently used between a robot and the HMD in particular. Based on this, authors proposed three referencing algorithms that can serve this particular domain: Semi-Automatic One Shot, Automatic One Shot, and Automatic Continuous. While the trials for the proposed automatic methods (based on neural networks) are still in their infancy, a detailed implementation of Semi-Automatic referencing (ICP and Super4PCS algorithms) was tested on a KUKA KR-5 robot. With a minimal user input - positioning a cube (a seed hologram) on the base of the robot and rotating its z-axis towards its front - the referenced robot will be augmented on the actual one via the Microsoft HoloLens display.

An AR teleoperation interface was implemented in Gradmann et al. (2018) of a KUKA lightweight robot using a Google Tango Tablet. The interface allows the user to change the robot joint configuration, move the tool center point, and perform grasping and placing objects. The application provides a preview of the future location of the robot by augmenting its corresponding virtual one according to the new joint configuration. Object Detection was done using Tango’s built-in depth camera and RGB camera and is based on DBSCAN algorithm.

The authors in (Chu et al., 2008.) used a Tongue Drive System as input for an assistive grasping system facilitated through an AR interface. The system implements the YOLO neural network [39] for object detection and a deep grasp algorithm (Chu and Vela, 2018) for detecting the graspable locations for each object. Consequently, this information (bounding boxes and grasp lines) will be properly augmented on objects within the user’s FOV. Furthermore, a virtual menu provides the user with robot affordances that can be performed.

### Aerial Robots

A teleoperation surveillance system was proposed in Sawarkar et al. (2016) composed of an unmanned ground vehicle (UGV) and an unmanned aerial vehicle (UAV) in the context of a hostile environment. The IMU measurements of a VR goggle are used to control the rotations of a camera mounted on each vehicle. The live video stream is processed to detect individuals and their probabilities of being terrorists using a CNN. This information is then augmented to the user through the goggle.

As implied in literature, artificial intelligence techniques are a great means for a robust visualization and an improved user experience. Traditional techniques to augment information on objects or targets are mainly using fiducial AR markers, which are impractical in cases of new environments such as in urban search and rescue (USAR) scenarios. On one hand deep learning can improve robot perception of its environment to detect objects and properly augment related information on each. On the other hand, it can be used to localize the robot itself and reveal information during its live performance. A key consideration for these systems is the processing requirements versus the current capabilities of the hardware.

### Application-Based Analysis

This section focuses on the areas in which AR and AI were applied. In other words, we explain here how the challenges of a certain robotics application - such as learning from demonstration and robot localization - were addressed through leveraging resources from augmented reality and artificial intelligence. We divide this into three main headings: Learning (12 papers), Planning (8 papers), and Perception (9 papers). Tables 3, 4, and 5 summarize the advantages as well as disadvantages and limitations of each method in each of the three subheadings respectively.

**TABLE 3:** The advantages as well as the disadvantages and limitations of each method in the Learning sub-heading.

References	Effects of AR and AI	
	Advantages	Disadvantages and limitations
Ong et al. (2010)	+ Intuitive visualizing and planning of EE orientations and CFV. +21.7% decrease in max error compared to the base case	- Performance deteriorates for non-planar surfaces due to poor tracking capabilities. - High speed of manual curve tracing can cause large variations
Fang et al. (2013)	+ Add, modify, and delete control points. + define EE orientation angles at control points	- Distracting AR visualizations. - Unexperienced users found difficulties in not occluding the marker
Fang et al. (2014)	+ User-friendly interphase to visualize and modify control points	- Performance deteriorates for non-planar surfaces due to poor tracking capabilities. - High speed of manual curve tracing can cause large variations
Warrier and Devasia, (2018)	+ 20% decrease in tracking errors. + User-friendly interface to demonstrate trajectories and visualize them	- Multiple trials are needed during the training phase to record the response dynamics. - Limited FOV of the HoloLens
Bentz et al. (2019)	+ Robot can learn human visual interest while avoiding collisions. + Recorded head motions reduced by 0.47s per subject	- Increase in human’s performance time by around 10–16 s
Liu et al. (2018)	+ Intuitive interface for adding a new task and removing an existing one. + Visualizing robot’s action plan as a T-AOG. + An early diagnosis of failure. + Increased success rate in robot opening bottles	- Error prone robot localization and tracking of markers. - Limited FOV provided by HoloLens - Images provided by HoloLens are blurred with a low-quality color contrast
Tay et al. (n.d.)	+ Detection of faulty conditions based on IMU data. + Decreasing the time spent on troubleshooting	- Poor localization ability
Dias et al. (2020)	+ Intuitive AR interphase for robot control. + 80% accuracy in performing a task variant	- Model predictions of invalid actions
Cao et al. (2019)	+ Intuitive interface for visualizing saved demonstrations as ghost holograms. + Editing demonstrations in real time	- Half of participants found AR ghosts to be distracting and obstructive after some time
Zein et al. (2020)	+ Intuitive interface for selection of suggested trajectories. + The average distance, time, and Hausdorff metric were improved by 14.8, 16.4, and 7.7% respectively	- Impractical using real drones: AR visualizations will only be applicable for a limited view of the drone (given the constrained FOV of HMDs or projection-based systems)
Gadre, (2018)	+ User-friendly interface for robot teleoperation. + Intuitive editing of endpoints. + Shadow visualizations of future orientation	- Unstable robot hologram (drifting due to poor tracking). - Error prone alignment of virtual robot with real robot, and sphere locations

**TABLE 4:** The advantages as well as the disadvantages and limitations of each method in the Planning sub-heading.

References	Effects of AR and AI	
	Advantages	Disadvantages and limitations
Muvva et al. (2017)	+ Robot successfully avoided both physical and augmented obstacles	- Impractical robot setup for some field applications. - Robot cannot avoid any new physical object
Chakraborti et al. (2017)	+ Enforcing safety to the H-R workspace. + Closed loop feedback through EEG signals	- Narrow FOV. - Non-robust robot performance: learning algorithm doesn’t always converge due to limited Emotive SDK capabilities
Corotan and Irgen-Gioro, (2019)	+ Intuitive interface for visualizing and controlling robot behavior. + Inexpensive setup: single device for localization, routing, and object detection	- Poor robot localization. - Camera obstructions cause major performance drops. - Motion tracking fails for long distances
Weisz et al. (2017)	+ A user-friendly interface for visualizing and accepting grasps. + Successful grasping of 82% of objects	- The grasp refinement stage is very time-consuming
Zhang et al. (2020)	+ Visualizing AR grasp overlays and planned grasp lines. + Success rate in grasping all 8 objects was 95%. + Can produce optimal grasps that the standard top-down approach can’t	- Scanning process is tedious and time consuming
(Chu et al., n.d.)	+ Visualizing an intuitive virtual menu for robot affordances. + The proposed pipeline increases operation speed by five times	- Success rate decreases by 20% compared to the manual method

**TABLE 5:** The advantages as well as the disadvantages and limitations of each method in the Perception sub-heading.

References	Effects of AR and AI	
	Advantages	Disadvantages and limitations
Ghiringhelli et al. (2014)	+ Visualizing perceived knowledge of each robot. + Adopted tracking algorithm works even on very small robots	- Unless two LEDS are mounted and visible on each robot, tracking fails
Sawarkar et al. (2016)	+ Allowing teleoperator to access and control a first person display of the remote environment. + Visualizing the algorithm’s results in detecting a terrorist	- Limited FOV of the HoloLens
Wang et al. (2018)	+ Localizing and tracing the object of a target in the robot’s environment	- Algorithm fails if target is not in the room. - Post-processing segmentation results is needed
Gradmann et al. (2018)	+ 82% of program executions were successful. + Intuitively change joint, robot control mode, and gripper control	- Slow object detection runtime. - Adopted algorithm might be inapplicable given a high number of objects in scene
Sprute et al. (2019b)	+ 91.5% F1 score for LSS. + Accuracy is as high as for a system without an LSS. + Easy validation of AI output	- Any occlusions to the overhead camera might cause failing. - Complicated setup in some environments. - Performance highly depends on the type of environment
Puljiz et al. (2019)	+ Minimizing human workload for calibration	- Decrease in robustness and accuracy compared to marker-based approaches
De Gregorio et al. (2020)	+ Intuitive interface to define and modify bounding boxes. + Diminishing the computational cost of image labeling. + Decreasing the annotation time by up to 9 h +15% increase in precision and recall scores	- The preliminary stage of the pipeline requires manual object detection, which might cause inaccuracies
Kästner et al. (2020)	+ Markerless 3D object localization. + 97% decrease in computational time	- 3% decrease in accuracy
Kastner et al. (2020)	+ Markerless 3D object localization	- High computational cost - Impractical and could result in a bad user-experience
El Hafi et al. (2020)	+ Teaching robot new spatial concepts through the AR interface and natural language. + Monitor AI status in real time	- Expensive solution for retail stores (given the current price of HMDs)

#### Learning

In general terms, a robot is said to learn from its environment or from the human if it can develop novel skills from past experience and adapt according to the situation at hand. According to the collected literature, we divide the scope of learning here to two basic paradigms: Learning from demonstration and Learning to augment human performance.

### Learning From Demonstration

Robot learning from demonstration (LFD) is described as the ability of a robot to learn a policy – identified as a mapping between the robot world state and the needed actions – through utilizing the dataset of user demonstrated behavior (Argall et al., 2009). This dataset is called the training dataset, and it is formally composed of pairs of observations and actions. Consequently, training channels are a bottleneck in such applications, and this is where augmented reality comes very handy. AR interfaces can act as a means for demonstrating the required behavior, and more importantly, improve the overall process through demystifying user intent. Consequently, the user can intuitively understand the “robot intent” (i.e., how the robot is understanding his/her demonstration). On the other hand, AI can be used for the robot to learn the “user intent” (i.e., understand what the user wants the robot to perform and adapt accordingly), and visualize this intent through AR. The following analysis clarifies this within the context of LFD.

In Ong et al. (2010) and Fang et al. (2013, 2014), data points of the demonstrated trajectory (of a virtual robot) are collected, edited, and visualized through a HMD/GUI allowing the user to intuitively clarify his/her intent of the desired trajectory. These demonstrations are first parameterized using a Piecewise Linear Parameterization (PLP) algorithm, then fed to a Bayesian neural network (BNN), and finally reparametrized. Authors compared error metrics and demonstrated that the proposed three-stage curve learning method (PLP, BNN, and reparameterization) improved the accuracy of the output curve much faster than the basic approach. Similarly, authors in Gadre (2018) used the Microsoft HoloLens as an interface for data collection in demonstrating a desired curve for a real Baxter robot. The interface allows the user to interactively control a teleoperation sphere augmented on the robot EE. The environment is modeled as a Markov Decision Process, and the agent (robot) learns a Dynamic Movement Primitive based on the user-defined critical points. Data from demonstrations were processed through a least-square function. Although this methodology supports an intuitive interface for collecting training data, it was prone to errors as the real robot and hologram were not lining up all the time, causing inaccurate representation of locations. Furthermore, the system was only tested by a single expert demonstrator.

In Warrier and Devasia (2018), the authors trained a kernel-based regression model to predict the desired trajectory of the EE based on a database of human-motor dynamics. Through observing the human-motor actions collected through a Microsoft Kinect camera, the model can infer the intent of the user of the desired trajectory. A single trial allows the robot to infer a new desired trajectory, which is then visualized to the user through the HoloLens against the actual demonstrated trajectory. This allows the user to spatially correct the error through moving their hand (tracked using the Skeleton Tracking routine) to minimize the distance between the demonstrated and desired trajectories. Alternatively, the authors in Liu et al. (2018) captured demonstrations by tracking hand-object interactions collected through a LeapMotion sensor. After manually segmenting the captured data into groups of atomic actions (such as pinch, twist, and pull), this data is used to train a modified version of the unsupervised learning algorithm: ADIOS (Automatic Distillation of Structure). This induces a Temporal and Or Graph (AOG), a stochastic structural model which provides a hierarchical representation of entities. The AR interface then allows to interactively guide the robot without any physical interactions, for example through dragging the hologram of the virtual robot to a new pose.

In Cao et al. (2019), the human motion is captured through the AR elements (Oculus Rift and two Oculus Touch Controllers) and saved as ghost holograms. Dynamic Time Warping is used to infer the human motion in real time from a previously compiled list of groups that represent human authorized motions. The workflow of the proposed system consists of five modes: The Human Authoring Mode in which the demonstrations are recorded, The Robot Authoring Mode in which the user can interactively author the collaborative robot task, The Action Mode in which the user performs the new collaborative task, and The Observation and Preview Modes for visualizing saved holograms and an animation of the whole demonstration.

A tablet was used in Dias et al. (2020) for data collection, prompting the user to construct a toy building through controlling a multi-robot system consisting of two mobile robots to carry blocks of different types and one robot arm for pick and place and a grid of state cells is used to represent the workspace. Given that the user can select between 135 possible actions to construct the toy, the application stores this data for training the DNN model. The model computes the posterior probability of the uncertain action (how the user is building the structure), predicting the move with the highest probability depending on what the current state is in the occupancy grid. Although the model performed successful task variants for 80% of the trials, authors indicated that further improvements should be done to improve the prediction of sequential actions and investigate more complex tasks.

### Learning to Augment Human Performance

Machine Learning opens a great avenue for improving the quality and efficiency of tasks performed by humans, such as maintenance and troubleshooting, multitasking work, or even teleoperation. AI would be used for understanding data and providing suggestions that would augment (improve) human performance of the task at hand. In the following analysis, we analyze content within this perspective, focusing on how the application was improved.

Multitasking is improved in Bentz et al. (2019), where data from a HMD are fit to a model that identifies views of interest to the human, directs an aerial co-robot to capture these views, and augments them on his/her. The input data is the head pose collected through a VICON motion capture system. A function, modeled as a mixture of Gaussians, receives this data and estimates the human visual interest via expectation maximization (EM). Although the average time to complete the primary task increased by around 10–16 s, the head motions recorded throughout the experiment were reduced by around 0.47 s per subject.

In Tay et al. (n.d.), the authors investigated two machine learning models trained on IMU sensor data of a Turtlebot to predict possible motor failures. SAS Visual Data Modelling and Machine Learning (VDMML) was used to test which of the Random Forest Model and Gradient Boosting would perform better to track the balance (tilting) of the robot. Gradient Boosting was chosen as it showed a lower average squared error in predictions, with 315 generated decision trees and 426 maximum leaf size.

An “Autocomplete” framework was proposed in Zein et al. (2020) that would support novice users in teleoperating complex systems such as drones. The system takes the human input from a Joystick, predicts what the actual desired teleoperation command is, and then shares it with the user through an augmented reality interface. The used model is an SVM trained on 794 motion examples to classify the input motion as one from a library of motion primitives which currently are lines, arcs, 3D helix motions, and sine motion.

In this section, two learning paradigms were discussed, robot learning from demonstration (LFD) and robot learning to augment human performance. The presented literature affirms that AR and AI will be extensively integrated in these two robotics applications in the near future. In the former, AR serves as a user-friendly training interphase and has a great potential for swarm mobile robotics, as multiple users can more easily train a multi-robot system. In the context of manipulators and robotic arms, visualizing demonstrations in real time allows the user to understand trajectories, correct for errors, and introduce new constraints to the system. In the latter, there is a potentially growing avenue to employ AI in robotic applications that understand user instructions (of the task at hand) and employ AR to visualize what the robot understands and interactively ask for feedback from the user. This has a great potential in complex applications where multiple factors concurrently affect the process, such as in the cases of teleoperating unmanned aerial vehicles (UAVs) or controlling mobile robots in dynamic environments like in the case of USAR.

#### Planning

This cluster groups papers in which AI is integrated to improve task planning, path planning, and grasping.

### Task Planning

In Chakraborti et al. (2017), a system for human-aware task planning was proposed featuring an “Augmented Workspace” allowing the robot to visualize their intent such as their current planning state, and a “Consciousness Cloud” which learns from EEG signals the intent of the human collaborator while executing the task. This cloud is two-fold: an SVM model is used to classify input EEG signals into specific robot commands, and a Q-learning model which learns from the task-coupled emotions (mainly stress and excitement levels) the preferences of the human to plan accordingly. Although results were promising on novice users, authors reflected that the significance of the system might drastically decrease when tested on experienced individuals and proposed this as a future work.

### Path Planning

Optimal path planning through reinforcement learning was done in Muvva et al. (2017) in a working environment combining both physical and AR (virtual) obstacles. The environment is represented as a Markov Decision Process, and the Depth First Search (DFS) was used for a sub-optimal solution. Then the robot is trained to find the optimal path in grid world using Q-learning which returns the path as the optimal policy learned. Similarly in Corotan and Irgen-Gioro (2019), the robot learns the shortest path to its destination using Q-learning while relying solely on ARCore capabilities of localization and object avoidance. However, authors concluded that the robot’s dependence on one input (basically the camera of a smart phone mounted on the robot) supported by ARCore is inefficient. Whenever anything obstructs the sensor, the robot loses its localization and routing performance.

### Grasping

A deep AR grasp planning system was proposed in Zhang et al. (2020) which utilizes the ARKit platform to collect point cloud data of the object-to-grasp as well as visualizing the planned grasp vector overlaid on the object’s depth map. The pipeline is five-folds: Recording RGB images of the object to grasp, extracting the point cloud using Structure from Motion (SFM), cleaning the data using RANSAC and KNN, transforming the data to an artificial depth map, and finally feeding this map to a pre-trained GQ – CNN. Although this methodology was efficient in detecting optimal grasps for cases where the traditional top-down approach fails, its downside is the very high time taken for collecting data (2 min per object).

The authors in Chu et al. (2018) also investigated AR and AI solutions for grasping, specifically those controlled by a Tongue Drive System (TDS). The input is RGB-D images from the META AR glasses, and the output is potential grasp predictions each represented by a 5D grasp rectangle augmented on the target object. Before applying the deep grasp algorithm (Chu et al., 2018), YOLO (Redmon et al., 2016) is first applied on the RGB-D for generating 2D bounding boxes, which are further manipulated into 3D bounding boxes for localization. The system achieved competitive results with state-of-the-art TDS manipulation tasks.

Through using grasp quality measurements in Weisz et al. (2017) taking into consideration the uncertainty of the grasp acquisition and the object’s local geometry in a cluttered scene, the system can robustly perform grasps that match the user’s intent. The presented human-in-the-loop system was tested on both healthy and impaired individuals and subjects successfully grasped 82% of the objects. However, subjects found some difficulties in the grasp-refinement phase mainly due to their lack of the gripper’s friction properties.

Based on the literature presented, we foresee several opportunities for the utilization of AR and AI in future planning and manipulation tasks. This can result in a paradigm shift in collaborative human-in-the-loop frameworks, where AI can add the needed system complexities and AR can bridge the gap for the user to understand these complexities. For example, the challenges of assistive robotic manipulators (Graf et al., 2004; Chen et al., 2013) to people with disabilities can be mitigated, and the integration of new input modalities to grasp planning can be facilitated. Concurrently, in all planning frameworks, attention should be given to the added mental load of AR visualizations, which might obstruct the user in some cases or even hinder efficient performance.

#### Perception

This cluster groups papers in which AI is integrated for robot and environment perception through object detection or localization.

### Object Detection

In Sawarkar et al. (2016) the data received from the IP camera mounted on the UGV is initially de-noised using the Gaussian filter, then processed using two algorithms for detecting individuals: an SVM trained with HOG features, and a Haar Cascade classifier. These algorithms detect the human anatomy and selects it as the ROI, which is then fed to a CNN trained to recognize individuals holding several types of guns. Once the data is processed, the detected human is augmented with a colored bounding box and a percentage representing his/her probability of being a terrorist.

In Wang et al. (2018), an automatic target detection mode was developed for the AR system based on an object semi-supervised segmentation applied to a convolutional neural network. The segmentation algorithm used is the One-Shot Video Object Segmentation (OSVOS). The methodology is limited as the chosen algorithm was prone to errors especially when there is no target in the view. Furthermore, post-processing the results was needed unless the user manually specifies whether a target is within view or not.

In De Gregorio et al. (2020), authors compared the results of two object-detecting CNNs: YOLO and SSD on the dataset they generated using ARS, an AR semi-automatic object self-annotating method. The proposed method enabled the annotation of nine sequences of around 35,000 frames in 1 hour compared to manual annotation which usually takes around 10 h to annotate 1,000 frames improving the data annotation process. Furthermore, both recall and precision metrics were increased by around 15% compared to manual labeling. In El Hafi et al. (2020), authors developed a method to form spatial concepts based on multimodal inputs from imaged features obtained by AlexNet-based CNN (Krizhevsky et al., 2012), self-location information from the Monte Carlo localizer, and word information obtained from a speech recognition system.

To reduce the time spent on restricting the workspace of mobile co-robots, authors in Sprute et al. (2019b) developed a learning and support system that learns from previous user-defined virtual borders and recommends similar ones that can be directly selected through an AR application. The system uses a perception module based on RGB cameras and applies a deep learning algorithm (ResNet101) to the semantically segmented images of previous user interactions. Some limitations are mainly due to occlusion from furniture or having a camera setup that doesn’t cover the whole area.

The DBSCAN algorithm was used in Gradmann et al. (2018) to detect objects for a pick and place task. Objects are clustered according to their depth and color information provided by the depth camera of the Google Tango tablet. AR provides a live visual interface of the detected objects and a preview of robot intent (future position). 82% of pick and place tasks with different object positions were performed successfully, although the algorithm’s runtime can be impractical for some applications.

### Robot Localization

In order to localize the robot and properly augment the information on each robot in a multi-robot system, authors in Ghiringhelli et al. (2014) used an active marker (one blinking RGB LED per robot) imaged by a fixed camera overlooking the robots environment. The blinking of each LED is set to a predefined pattern alternating two colors (blue and green). Initially, bright objects were detected through a fast beacon-detection frame-based algorithm. These detected objects were filtered first through evaluating the Track Quality Index, and then through a *linear binary* model which classifies the tracked points of the RGB color into either blue or green, based on a *logistic regression* learning of the blue and green color features applied during calibration.

The authors in Puljiz et al. (2019) presented a review of different approaches that can potentially be used for referencing between a robot and the AR HMD, such as training a neural network to estimate the joint positions of a robot manipulator based on RGB data (Heindl et al., 2019). This was actually done in Kästner et al. (2020) to localize the six DOF pose of a mobile robot instead, while evaluating the training process through the AR interface. Authors compared two state-of-the-art neural networks – SSPE and BetaPose - previously trained on real and artificial datasets. The artificial dataset is based on a 3D robot model generated by Unreal Engine and annotated using *NDDS* plugin tool. Both networks, upon receiving a live video stream from the HoloLens, predicted accurate 3D pose of the robot, with the SSPE being 66% faster. Estimating the pose based on depth sensor data was investigated in Kastner et al. (2020). Authors also developed an open source 6D annotation tool for 2D RGB images.

In this section, almost all the literature is an integration of AI to improve the AR experience, whether in innovating robust calibration methods or improving the tracking and object detection capabilities of AR systems. This provides an insight of what is done and what can be done to achieve a smooth integration of augmented reality applications. These methods are still limited in terms of robustness to ambient conditions like lighting, and the problem of increased computational time is still impractical for some applications. However, this can be mitigated in the future as hardware power is constantly improving and cloud computing is becoming ubiquitous.

### The Ethical Perspective of Robotics and AI

As robots become ubiquitous, there are ethical considerations ranging from liability to privacy. The notion of a robot’s ability to do ethical decision making was first framed in Wallach and Allen (2009) yet the need to set rules for robot morality has been foresighted much earlier in Asimov’s fiction literature. Several organizations are trying to set guidelines and standards for such systems, we mention the IEEE 7010–2020 standard on ethically aligned design. The ethical challenges arising from complex intelligent systems span civilian and military use. Several aspects of concern emerged, ranging from discrimination and bias to privacy and surveillance. Service robots, which are designed to accompany humans at home or work present some of the greatest concerns as they serve in private and proprietary environments. Currently, AI capabilities possessed by robots are still relatively limited, where robots are only capable of a simple navigation task or taking a simple decision. However, as the research field evolves, robots will be able to do much more complex tasks with a greater level of intelligence. Therefore, there is a moral obligation for ethical consideration to evolve with the evolving technology.

## Concluding Remarks

This paper provided a systematic review of literature on robotics which have employed artificial intelligence (AI) algorithms and augmented reality (AR) technology. A total of 29 papers were selected and analyzed within two perspectives: A theme-based analysis featuring the relation between AR and AI, and an application-based analysis focusing on how this relation has affected the robotics application. In each group, the 29 papers were further clustered based on the type of robotics platform and the type of robotics application, respectively. The major insights that can be drawn from this review are summarized below.

Augmented reality is a promising tool to facilitate the integration of AI to numerous robotics application. To counter the effect of increased complexity in understanding AI systems, AR offers an intuitive way of visualizing the robot internal state and its live training process. This is done through augmenting live information to the user via an HMD, a desktop-based GUI, a mobile phone, or a spatial projection system. This proved to improve several applications, such as learning by demonstration tasks, grasping, and planning. Learning from demonstration for robot manipulators is a field that has greatly benefited from the integration of AR and AI for an intuitive and user-friendly method of teaching, as done in Fang et al. (2014) and Liu et al. (2018). AR has served as a user-friendly interface to ask the user to accept or reject the AI output, such as recommending to “Autocomplete” a predicted trajectory or suggesting a faster mapping of new virtual borders. We suspect the use of AR could contribute to the acceptability and trust of the general public in AI-enabled robots, as it can explicitly reveal the decision-making process and intentions of the robot. This also has the potential to contribute to not only increasing the efficiency of robotic systems but also their safety.

To improve the AR experience, accurate and reliable calibration and object localization methods are needed. As can be seen from the literature, artificial intelligence is a viable element supporting this notion for robotics applications. AR markers are widely used but are limited in dynamic environments and in cases of occlusions. Deep neural networks for object detection and robot localization seem the most promising for unstructured robotic environments (De Gregorio et al., 2020; El Hafi et al., 2020), although they rely more on computational power and some methods are still computationally demanding. However, progress in hardware and cloud computing is making AI more viable in such scenarios. We suspect that AI will be used more for context and situational awareness in addition to detection of objects and events, which are capabilities that would enrich more AR displayed content.

The potentials of integrating these two elements in robotics applications are manifold and provide a means of deciphering the traditional human-robot mismatch model. Specifically, in the context of human-robot collaboration, AI can be used to understand the real user intent filtered from the perceived tasks the robot traditionally performs as in the work of Zein et al. (2020). At the same time, AR can visualize information of the robot’s understanding of the user intent as in the work of Ghiringhelli et al. (2014), providing a closed feedback loop into the model mismatch paradigm. The combination of these technologies will empower the next phase on human-robot interfacing and interaction. This is an area that highlights the importance of AI working side by side with humans instead of being perceived as a substitute for them.

This study confirms the many benefits of integrating AR and AI in robotics and reveals that the field is fertile and expects a striking surge in scholarly work. This result aligns with the current trends of incorporating more AI in the field of robotics (Dimitropoulos et al., 2021). After the outbreak of COVID-19, the demand to replace humans with smart robots have become critical in some fields (Feizi et al., 2021) affirming the increasing trend. Similarly, AR technology is currently at its rise, with several broad applications spanning education (Samad et al., 2021), medicine (Mantovani et al., 2020), and even sports (da Silva et al., 2021). As AR and AI related technologies evolve, their integration will have numerous advantages to every application in robotics as well as other technological fields.”

Despite the well-developed resources, some limitations need to be addressed for powerful implementation of AR and AI in robotics. For example, AR devices are still hardware-limited, and some do not support advanced graphical processing, which challenges the implementation of computationally intensive AI algorithms on AR devices in real-time. Current methods rely on external remote servers for heavy computations, which might be impractical in some cases. Furthermore, vision-based approaches to track objects using AR markers are prone to errors and performance drops largely when occlusions happen or under challenging lighting conditions. Further improvements in AR hardware are needed to improve processing, battery life, and weight; all are elements needed for AR use for an extended period of time.

Future work can apply new out-of-the-box AItbox1 techniques to improve the AR experience with tracking methods robust in dynamic situations. Additional work is needed in AI to better understand human preferences in “how,” “when,” and “what” AR visual displays are shown to the user while debugging or performing a collaborative task with a robot. This can be framed when a robot can fully understand the “user intent” and show the user only relevant information through an intuitive AR interface. Similarly, AR holds potentials for integrating AI in complex robotics applications, such as grasping tasks in highly cluttered environments, detecting targets and localizing robots in dynamic environments and urban search and rescue, and teleoperating UAVs applying intelligent navigation and path planning. The future will have AI and AR in robotics ubiquitous and robust, just like networking, a given in a robotic system.

The major limitation of this systematic review is the potential underrepresentation of some papers combining AR, AI, and robotics. Given the choice of search terms identified in *Methods*, there is a possible incomplete documentation of research papers that do not contain a specified keyword, rather contain another synonym or an implied meaning in text.

## Data Availability

The original contributions presented in the study are included in the article/Supplementary Material, further inquiries can be directed to the corresponding author.

## Abbreviations

AR, Augmented Reality; MR, Mixed Reality; AI, Artificial Intelligence; HMD, Head Mounted Display; GUI, Graphical User Interface; PBD, Programming by demonstration; OGM, Occupancy Grid Map; CNN, Convolutional Neural Network; FOV, Field of View; KNN, K-Nearest-Neighbor; SVM, Support Vector Machine; EEG, Electroencephalographic; SFM, Structure from Motion; RANSAC, Random Sample Consensus; CNN, Convolutional Neural Network, YOLO, You Only Look Once; SSD, Single Shot Detector; ADIOS, Automatic Distillation of Structure; RGB, Red Green Blue; SVD, Singular Value Decomposition; MDP, Markov Decision Process; DTW, Dynamic Time Warping; EE, End Effector; DMP, Dynamic Movement Primitive; CP, Critical Point; ROS, Robot Operating System; GQ – CNN, Grasp Quality CNN; UGV, Unmanned Ground Vehicle; DBSCAN, Density-Based Spatial Clustering of Applications with Noise.
